# Understanding the Stringent Response: Experimental Context Matters

**DOI:** 10.1128/mbio.03404-22

**Published:** 2023-01-10

**Authors:** Jonathan Dworkin

**Affiliations:** a Department of Microbiology and Immunology, College of Physicians and Surgeons, Columbia University, New York, New York, USA; Ohio State University

**Keywords:** (p)ppGpp, growth arrest, nutrient limitation, quiescence, starvation

## Abstract

As rapidly growing bacteria begin to exhaust essential nutrients, they enter a state of reduced growth, ultimately leading to stasis or quiescence. Investigation of the response to nutrient limitation has focused largely on the consequences of amino acid starvation, known as the “stringent response.” Here, an uncharged tRNA in the A-site of the ribosome stimulates the ribosome-associated protein RelA to synthesize the hyperphosphorylated guanosine nucleotides (p)ppGpp that mediate a global slowdown of growth and biosynthesis. Investigations of the stringent response typically employ experimental methodologies that rapidly stimulate (p)ppGpp synthesis by abruptly increasing the fraction of uncharged tRNAs, either by explicit amino starvation or by inhibition of tRNA charging. Consequently, these methodologies inhibit protein translation, thereby interfering with the cellular pathways that respond to nutrient limitation. Thus, complete and/or rapid starvation is a problematic experimental paradigm for investigating bacterial responses to physiologically relevant nutrient-limited states.

## INTRODUCTION

Experiments in the early 1950s demonstrated that Escherichia coli exhibits reduced synthesis of nucleic acids upon starvation for an amino acid (1, 2). These seminal observations and many subsequent studies used a strain auxotrophic for an amino acid and subjected it to complete starvation for that amino acid. Stent and Brenner called this “stringent amino acid control” and identified a mutation (*rel*) whose phenotype—“relaxed”—was a loss of this coupling for all amino acids (3). Cashel and colleagues discovered a molecule, “magic spot,” that appeared during amino acid starvation and was absent in the *rel* mutant strain (4). Subsequently, they identified this molecule as guanosine tetraphosphate (ppGpp) (5). The *rel* locus was further defined as encoding the RelA protein, a ribosome-associated ppGpp synthase that is activated by the presence of an uncharged tRNA in the ribosome A site (6).

E. coli RelA is a member of the long RSH (RelA/SpoT family) protein family that contain a N-terminal enzymatic domain (NTD) and a C-terminal regulatory domain (CTD) (7). The NTD exhibits both (p)ppGpp hydrolysis and (p)ppGpp synthesis activities, although in some bacteria (e.g., E. coli) the hydrolysis activity is cryptic (7). The CTD interacts with the deacylated tRNA in the A-site and with rRNA, resulting in allosteric stimulation of the NTD synthetic activity (8). In addition, the region containing the hydrolytic activity directly inhibits the synthetic activity, and this coupling is itself allosterically regulated by (p)ppGpp (9). This feedback presumably serves to optimize RelA function *in vivo*, but this has not been demonstrated.

The extent of tRNA charging reflects aminoacylation activity and amino acid availability and manipulations of either parameter affect ppGpp synthesis. For example, amino acid availability can be modulated by starvation of an essential amino acid in an auxotroph (2), downshift from an amino-acid-replete medium to an amino-acid-free medium (10), isoleucine starvation caused by excess valine (11, 12), or limiting isoleucine concentrations (13). Inhibition of tRNA charging results from the addition of an amino acid analog that is a direct competitor of the cognate tRNA synthase such as serine hydroxamate (14), direct inhibition of the aminoacyl tRNA charging reaction by mupirocin (15), which prevents tRNA^Ile^ charging by isoleucyl-tRNA synthetase, or use of a temperature-sensitive allele of valine-tRNA synthetase at the nonpermissive temperature (5). We focus here on (p)ppGpp synthesis stimulated by one of these methods, although, importantly, (p)ppGpp is also synthesized during conditions (e.g., nutrient exhaustion [13, 16] or diauxic shift [17]) even in the absence of explicit amino acid starvation or inhibition of tRNA charging. These methods, referred to here as stringent response (SR) stimulation have certainly facilitated characterization of important aspects of (p)ppGpp regulation, including a detailed molecular understanding of RelA function. However, their numerous indirect physiological effects obscure proper characterization of the direct physiological effects of (p)ppGpp synthesized by RelA.

## EFFECTS OF AMINO ACID STARVATION INDEPENDENT OF RelA ACTIVATION

In addition to stimulating (p)ppGpp synthesis, SR stimulation methods affect protein synthesis by depriving the ribosome of an essential substrate—a charged tRNA. Thus, they function, at least in part, to inhibit translation. For example, both direct amino acid starvation (18) or exposure to serine hydroxamate (14) reduce protein synthesis by >10-fold. The speed of this decrease, presumably similar to the time scale of (p)ppGpp synthesis in response to these treatments—within minutes—is faster than the temporal dynamics of growth slowdown following departure from exponential growth, the so-called “transition phase” that lasts for a few hours for E. coli grown in Luria-Bertani (LB) medium (19). Of note, growth arrest is substantially slower under isoleucine depletion (~100 min [13]), which may make it a useful method for studying more gradual transitions. Finally, while protein degradation can serve as a source of amino acids in starved E. coli (20, 21), the relatively slow kinetics of this process (22) suggests that it is likely not sufficient to counteract acute effects on translation.

**FIG 1:**
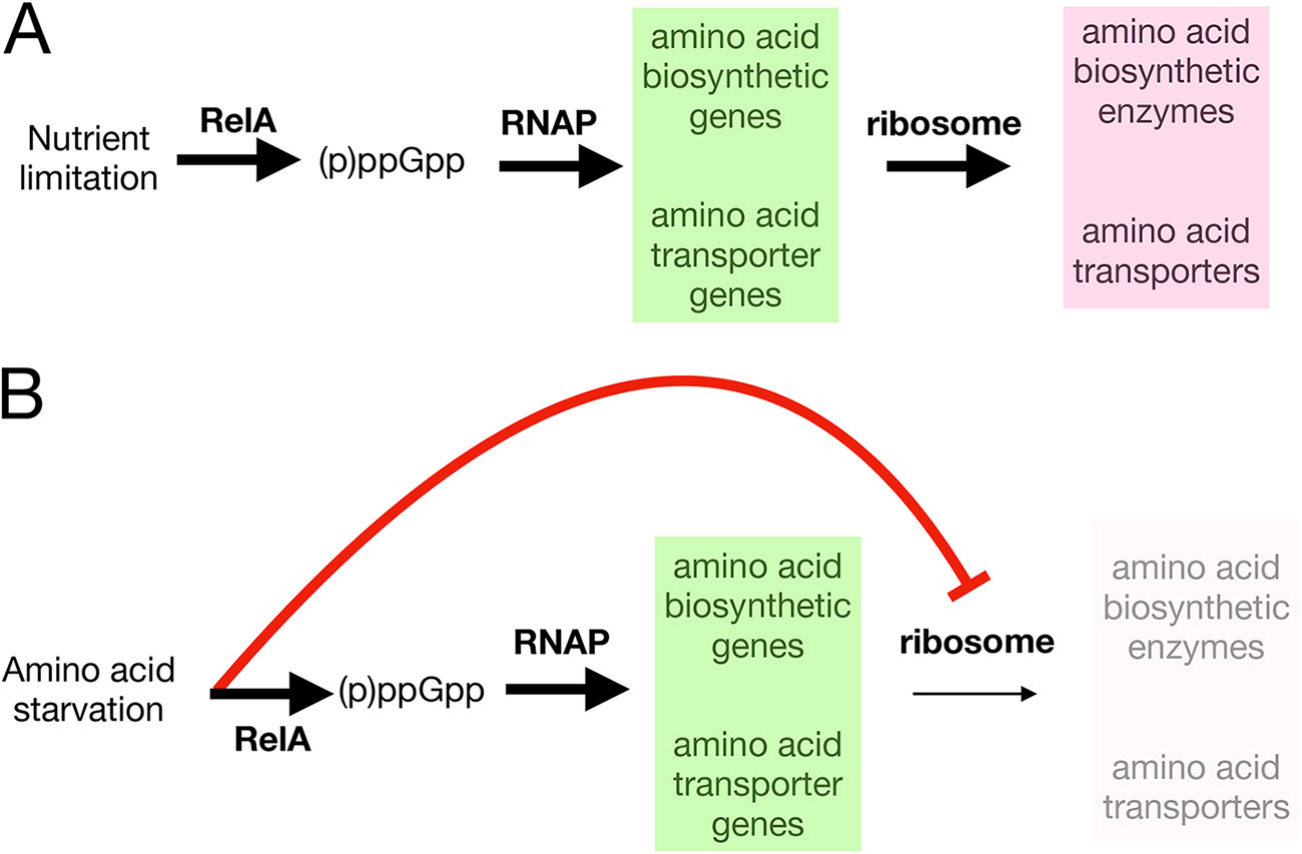
Effect of stringent response stimulation on compensatory protein synthesis. (A) Under conditions of nutrient limitation, ppGpp synthesized by RelA stimulates the transcription (green) of many genes, including those encoding amino acid biosynthetic enzymes and transporters. The mRNAs are then translated by the ribosome (pink). (B) During amino acid starvation, ppGpp again stimulates specific gene transcription, but the absence of amino acid(s) (red) prevents translation of the mRNAs.

Bacterial responses to nutrient limitation include *de novo* synthesis of specific proteins (23, 24), so translation inhibition caused by SR stimulation would obscure a direct effect of (p)ppGpp on translation (25). This inhibition likely would hamper the ability of cells to initiate compensatory responses to amino acid starvation that include *de novo* synthesis of amino acid transporters and biosynthetic enzymes (26, 27), consistent with the robust homeostasis of amino acid levels across a wide variety of physiological conditions (28). Specific examples of such responses include the RelA-dependent induction of the E. coli *his* operon under conditions of histidine starvation (29) and the many amino biosynthetic genes that are rapidly induced following overexpression of a truncated RelA (30). Addition of arginine hydroxamate to B. subtilis stimulates transcription of amino acid biosynthetic genes, including *ilvB* and *leuC*, but not in a strain missing all (p)ppGpp synthases, including *relA* (27). Moreover, consistently, ppGpp-dependent riboswitches are seen upstream of many branched-chain amino acid biosynthetic and transporter genes in *Firmicutes* (31). Since all of these mechanisms begin with gene activation but ultimately depend on protein expression, the inhibitory effects of SR stimulation on translation would substantially impair compensatory function (Fig. 1). Importantly, this is to be distinguished from any direct effect of (p)ppGpp on translation since this inhibition occurs after (p)ppGpp synthesis not before.

In addition to these compensatory responses to amino acid starvation, protein synthesis is critical for transitions in bacteria coupled to decreased nutrient availability. For example, initiation of replication in most bacteria is dependent on accumulation of the DnaA initiator protein. The rate of accumulation is dependent on both degradation and synthesis (32), and decreasing nutrient levels affect the rate of DnaA translation (33). While an inducible truncated RelA blocks replication initiation, ectopic synthesis of DnaA rescues this block (34). Thus, in this case, inhibition of protein synthesis resulting from SR stimulation would aberrantly block DNA replication.

By definition, prototrophic bacteria can grow in the absence of amino acids. Nevertheless, downshift from an amino-acid-replete to an amino-acid-free medium results in an extended period of nongrowth (10), accompanied by metabolic remodeling (35), as well as presumably substantially reduced protein synthesis that could have regulatory implications. For example, transcription factors, typically low-abundance proteins, may be particularly sensitive to changes in rates of synthesis with possible broad consequences for overall physiology (36). Consistently, l-valine-induced isoleucine starvation induces regulatory responses beyond the expected ppGpp regulon (37). Thus, treatments that stimulate (p)ppGpp synthesis and that also directly attenuate protein synthesis may not be appropriate proxies for nutrient limitation. Some of these issues may be specific to enteric bacteria since amino acid starvation is not sufficient to stimulate ppGpp accumulation in Caulobacter crescentus, a bacterium that lives in oligotrophic environments characterized by low amino acid concentrations (38).

Other essential processes in a growing cell depend directly on amino acid availability. For example, E. coli peptidoglycan consists of glycan strands cross-linked by a stem pentapeptide (l-alanine, d-glutamic acid, *meso*-diaminopimelic acid [*m*-DAP], d-alanine, and d-alanine). Since E. coli contains ~5 × 10^6^ stem peptides/cell (39), similar to the concentration of the *m*-DAP precursor aspartate (~10^7^ molecules/cell) (40), peptidoglycan synthesis should be quite sensitive to aspartate availability. In fact, aspartate starvation quickly abrogates peptidoglycan synthesis, with resulting effects on cell wall stability and the subsequent activation of cell wall stress pathways (41). Effects of amino acid starvation may be quite broad, since amino acid catabolism provides a large portion of the carbon and nitrogen in conditions of rapid growth (42). In addition, levels of branched-chain amino acids play a central role in a global response to nutrient limitation in Gram-positive species that is, at least in part, independent of ppGpp (43). Thus, in summary, SR stimulation that results in rapid amino acid starvation (e.g., downshift to a medium lacking amino acids) may very well impact other physiological processes, thereby complicating identification of the direct effects of these treatments.

## AMINO ACID STARVATION IN AUXOTROPHS

As discussed above, depriving an auxotroph of its required amino acid results in SR stimulation. Of note, this starvation occurs eventually even for auxotrophs grown in LB medium, since tryptone, its major constituent, is a mixture of amino acids whose catabolism is the carbon source (19). While auxotrophies are certainly useful in the laboratory, a bioinformatic survey of >1,300 Gram-negative strains predicted only ~50 to be amino acid auxotrophs. In fact, this number is likely an overcount (44) since a detailed analysis of predicted auxotrophs in another study identified alternative biosynthetic pathways in nearly all cases (45). Thus, complete amino acid starvation in the growth medium is unlikely to be an important proximal signal of nutrient limitation. However, amino acid biosynthesis is a central metabolic activity, and as such, reflects the energetic capacity of the cell. Thus, amino acid limitation arising from reduced synthesis could serve to signal general energetic constraints, at least for prototrophs.

Although perhaps rare, amino acid auxotrophies are not simply a laboratory convenience. For example, a methionine requirement is common among Pseudomonas aeruginosa strains isolated from cystic fibrosis patients (46). Auxotrophies may confer a fitness advantage *in vivo* since synthesizing amino acids is more energetically costly than acquiring them from the host (47) or from other members of a microbial consortium. Alternatively, auxotrophs would likely have higher (p)ppGpp levels due to limiting levels of host-acquired amino acids and since increased (p)ppGpp levels are associated with enhanced tolerance to antimicrobials (48), auxotrophies might facilitate persistence of both pathogenic and commensal species.

## tRNA CHARGING

RelA senses amino acid starvation by monitoring depletion of aminoacylated pools, that is, not the amino acids themselves, but rather the charging of tRNAs (49), since an uncharged tRNA in the ribosome A site is sufficient for ppGpp synthesis (6). More recent structural analysis of RelA bound to the ribosome revealed a structural transformation dependent on the aminoacylation state of the A site tRNA that stimulates (p)ppGpp synthesis (50–52). Therefore, a simple model is that changes in tRNA charging resulting from changes in amino acid abundance should affect (p)ppGpp synthesis. Consistently, excess amino acids prevent (p)ppGpp accumulation under glucose limitation (38) and inhibit the effect of RelA on a diauxic shift (17), suggesting that accumulation of uncharged tRNAs can function as a starvation signal.

Although amino acid starvation leads to a large (>10-fold) and rapid (~5 min) decrease in the charging of the cognate tRNA (53), how smaller and more gradual changes in global uncharged tRNA levels, as might be expected during nutrient limitation, affect RelA activation is not clear. A substantial concentration of uncharged tRNAs exist even under nutrient-excess conditions; the native uncharged fraction has been estimated to be 10 to 50% for most tRNAs in B. subtilis (54) or ~25 to 50% for several E. coli tRNAs (e.g., tRNA^Ala^, tRNA^His^, and tRNA^Thr^ [53]), thus potentially impairing detection of modest changes. The charging levels of specific tRNAs may, however, have important regulatory effects, as seen in the example of T-box control of amino acid biosynthetic operons in Gram-positive species (55). In the particular case of E. coli RelA, fatty acid starvation for cells grown in minimal medium leads to depletion of lysine that, in turn, leads to the accumulation of uncharged tRNA^Lys^ and activation of RelA (56), although fatty acid starvation in other species is sensed independent of RelA (57).

Amino-acyl tRNA synthases (aaTS) mediate tRNA charging and their levels are thought to be optimized for maximal growth. As mentioned above, addition of the isoleucyl-aaTS inhibitor mupirocin results in SR stimulation. However, mupirocin treatment of E. coli leads to a near absolute (~90%) deacylation of tRNA^Ile^ (58), a substantially greater effect than observed during nutrient limitation (54). In addition, mupirocin acts rapidly, inducing maximal ppGpp in under 5 min (58, 59). Thus, the kinetics and magnitude of the mupirocin effect may obscure the identification of the pathways that respond to amino acid limitation. A second method to interfere with tRNA charging, and thereby mimic amino acid starvation, is the use of an amino acid analog such as serine hydroxamate, a competitive inhibitor of serine-aaTS (14). However, serine hydroxamate itself may have physiological effects other than RelA stimulation. For example, inhibition of tRNA^Ser^ charging as a result of serine starvation, prevents translation of a transcription factor that regulates B. subtilis biofilm formation (60). Finally, under starvation for a single amino acid, the charged levels of its cognate tRNAs drop 10- to 40-fold (61). Thus, under all three of these conditions, tRNA charging dynamics likely differs from that found in nutrient limitation.

**FIG 2:**
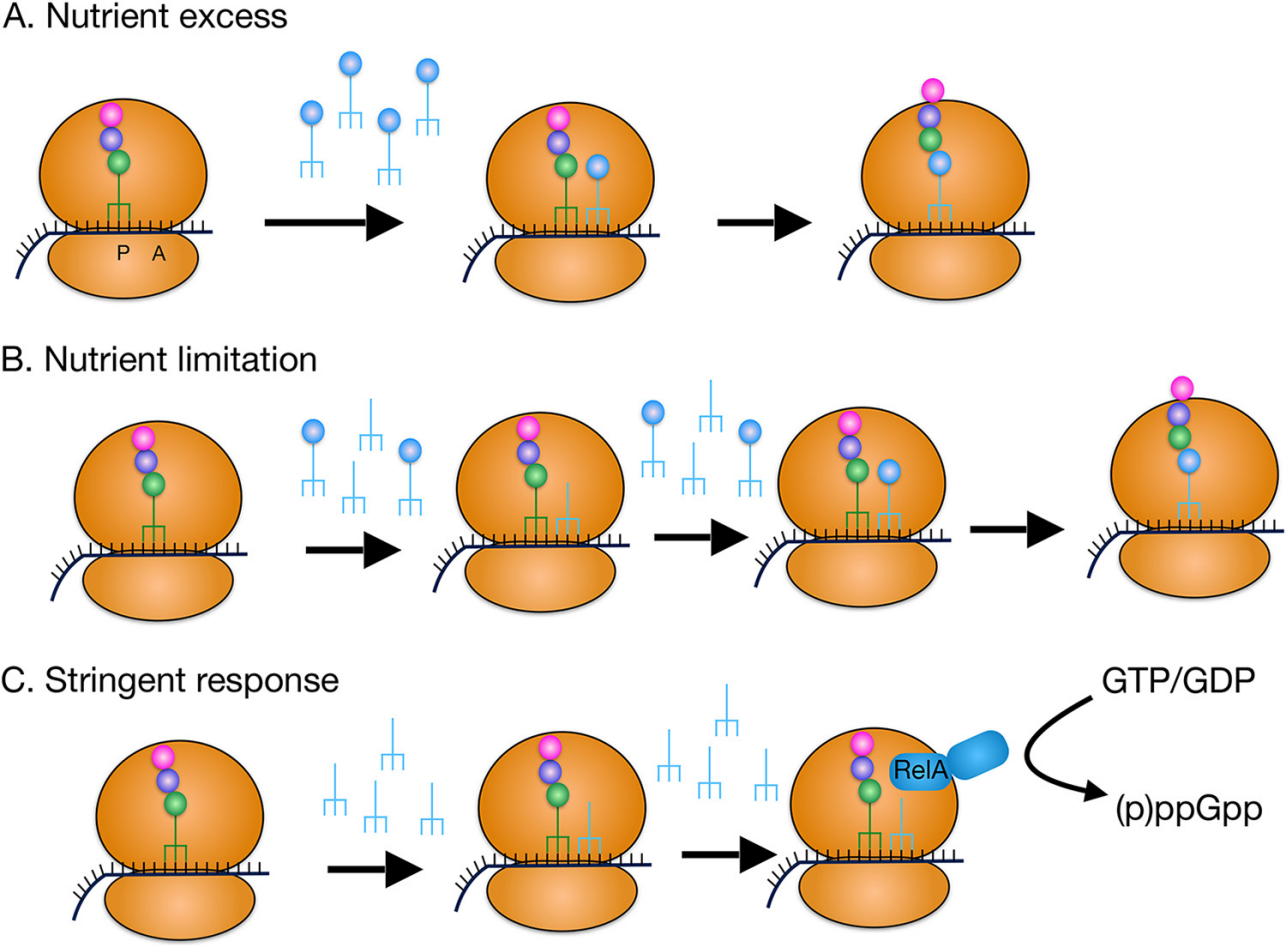
Effect of stringent response stimulation on translation. (A) Under conditions of nutrient excess, tRNAs are extensively charged, enabling efficient polypeptide chain elongation. (B) Under conditions of nutrient limitation, the tRNA pool is only partially charged, but even if an uncharged tRNA enters the A site, it can exit and be replaced by a charged tRNA molecule. (C) During SR stimulation, inhibition of tRNA charging (e.g., by the tRNA^Ile^ synthase inhibitor mupirocin) results in a substantial pool of uncharged tRNA^Ile^. Even though a given uncharged tRNA has a limited dwell time in the A site, it can only be replaced by another uncharged tRNA and polypeptide chain elongation is blocked. At the same time, RelA (p)ppGpp synthase activity is stimulated.

Diminished tRNA charging has important consequences for ribosome function. tRNAs have a defined dwell time in the A site so the presence of a minority of uncharged tRNA species would not be expected to interfere since an uncharged tRNA likely would be replaced by a charged tRNA (Fig. 2). In fact, uncharged tRNAs have a shorter dwell time compared to charged tRNAs (62). However, when uncharged species predominate (as under SR stimulation), the short dwell time may not be sufficient to prevent interruptions in translation. Also, the affinity of EF-Tu is greatly affected by the tRNA aminoacylation status; the affinity for deacylated tRNAs is ~1,000-fold lower than for acylated tRNAs (63). Thus, the essential function of EF-Tu in tRNA delivery to the ribosome A site may be substantially impaired under a regime of near complete tRNA deacylation.

Diminished tRNA charging underlies translational pausing, a reversible regulatory mechanism in bacterial and eukaryotic systems subject to amino acid limitation (64). However, even moderate deacylation can cause ribosome stalling (65), so total or near-total deacylation resulting from SR stimulation would likely cause extensive ribosome stalling. This could result in “ribosome traffic jams” or collisions (66), pathological conditions that may not be easily reversible and would likely have broad physiological implications. For example, ribosome collisions resulting from stalling (in fact induced by mupirocin [67]) lead to the formation of so-called “disomes” and the recruitment of exonucleases and subsequent mRNA cleavage (68). Thus, SR stimulation by inhibition of tRNA charging may pleiotropically affect the mRNA pool.

## KINETICS AND MAGNITUDE OF (p)ppGpp SYNTHESIS UNDER SR STIMULATION

RelA activation results in the synthesis of pppGpp and ppGpp from GTP and GDP, respectively. Since both molecules potentially affect central cellular processes such as translation, transcription, and ribosome assembly, understanding the kinetics and magnitude of their synthesis under different regimes of RelA activation is important. A detailed comparison of changes in GTP and ppGpp following SR stimulation by mupirocin with those observed during slowdown of growth indicates that these dynamics are different (59). GTP levels fall modestly as the culture growth slows (similar to previous reports [69]), whereas GTP levels fall substantially and ppGpp levels rapidly rise within minutes of mupirocin treatment in E. coli (59).

Such differences in the magnitudes of ppGpp accumulation may be biological significant. Specifically, many high-affinity ppGpp targets are GTP-binding proteins, often with substantial overlap in ppGpp and GTP binding sites (e.g., IF2 [70], Obg [71], RbgA [72]), and ppGpp acts as an orthostatic inhibitor in these cases. Direct competition between GTP and ppGpp implies that the intracellular concentration of GTP affects the fraction of ppGpp that is bound (73). Since concentrations of GTP fall much more following mupirocin treatment than during growth, the GTP/ppGpp ratio following mupirocin stimulation (~0.4) substantially deviates from that observed during nutrient limitation (~1.6) (59). These differences complicate interpretations of occupancy of GTP binding sites by (p)ppGpp. For example, EF-G is a proposed target of ppGpp (74), and its affinities for GTP and ppGpp are similar, 16.1 and 13.9 μM, respectively (75). Thus, changes in the ratio such as those induced by mupirocin may artifactually accentuate the ability of ppGpp to inhibit EF-G *in vivo*.

## IMPLICATIONS FOR UNDERSTANDING (p)ppGpp PHYSIOLOGY

The nature of the SR stimulation methods can affect investigations of the physiological role of (p)ppGpp. For example, translational GTPases in protein translation have long been known to be high-affinity *in vitro* targets of (p)ppGpp. However, surprisingly, the *in vivo* relevance of these measurements was not known even though protein synthesis is a straightforward parameter to monitor. The absence of *in vivo* data was likely a consequence of the SR stimulation methods that, as discussed above, directly impair protein synthesis. During nutrient limitation, a situation where (p)ppGpp is synthesized without explicit interference with translation, cells lacking RelA exhibit substantially increased protein synthesis (76), consistent with long-held assumptions.

Studies of RelA-mediated (p)ppGpp regulation typically employ one of the methods of SR stimulation described above (8, 77, 78). However, the pleiotropic indirect effects of these methods as discussed here suggest the utility of alternative methods to stimulate (p)ppGpp accumulation that, at least in theory, avoid these issues. These include ectopic expression of a truncated RelA mutant protein (30, 79, 80) or of a nonribosomal SAS synthase (81). However, RelA is subject to positive allosteric activation by its product (9, 82), so RelA overexpression and subsequent (p)ppGpp synthesis may be ultrasensitive to inducing conditions and may not reflect the kinetics of endogenous RelA activation. Thus, these methods should be properly calibrated to match (p)ppGpp accumulation in a physiological context such as slowing growth (59). For example, starvation of isoleucine over >100 min results in robust RelA-dependent ppGpp accumulation (13), and RelA is required for protein synthesis attenuation in the late transition phase (83). In summary, if (p)ppGpp functions analogously to an automobile brake (84), it certainly matters to the occupants of the car whether the driver slows down by slamming on the brakes or by more gingerly pumping the brake pedal!

## CONCLUSIONS

This discussion has pointed to numerous ways that SR stimulation methods have indirect effects that may interfere with identifying the direct effects of RelA and its product (p)ppGpp. Of particular relevance are the kinetics and magnitude of amino acid starvation at least in comparison between exhaustion (into stationary phase) and explicit starvation in the laboratory. This comparison is complicated since the question of exhaustion is not simple: careful attention to the relative concentrations of nitrogen and carbon sources in the growth medium is necessary to define the stoichiometrically limiting nutrient (85). For example, RelA affects translation under conditions of nitrogen limitation but not under carbon or phosphorus limitation (86). Moreover, of course, while nutrient limitation may be gradual in many contexts (87), physiological situations exist where starvation is abrupt and/or drastic, such as the transition of E. coli from the mammalian gastrointestinal tract to aquatic and soil environments that may be nutrient limited (88).

The experimental approaches used to stimulate ppGpp synthesis by activating RelA, as detailed above, have identified many important aspects of the central role played by RelA in ppGpp metabolism. However, RelA plays an important role in a variety of physiological contexts without explicit manipulation of amino acid levels. For example, the cyanobacterium Synechococcus elongatus synthesizes (p)ppGpp in response to the transition from light to dark. This RelA-dependent synthesis plays a key role in mediating broad physiological changes, including the inhibition of DNA replication and decreased rates of transcription and translation, and cells lacking RelA have greatly reduced viability in constant darkness (89, 90). While the absence of light results in RelA stimulation, the mechanism underlying this effect remains mysterious. *S. elongatus* RelA is similar to E. coli RelA, suggesting that it also is sensitive to an aminoacylation state, but this is not known. Another example is the phototroph R. palustris that can exist for weeks in a state of nongrowth following depletion of the carbon source. Entry into this state is accompanied by synthesis of (p)ppGpp by the single RelA-like Rsh protein in this organism, and a mutant lacking this protein quickly loses viability. Mycobacterium tuberculosis survives in a growth-arrested state with limited loss of viability over extended periods both *in vitro* and *in vivo* (91). Rel_Mtb_ synthesizes (p)ppGpp upon entry into stationary phase in response to hypoxia (92) or oxidative stress (93). The absence of Rel_Mtb_ results in reduced survival both *in vitro* during nutrient depletion and in the host. Caulobacter crescentus exhibits a stereotyped pattern of differentiation during its cell cycle that results in the production of a nonmotile stalked cell and a motile swarmer cell that subsequently differentiates into a stalked cell. Increased levels of (p)ppGpp synthesized by the single bifunctional RSH protein attenuates the ability of C. crescentus to proceed in the swarmer-to-stalked transition in the presence of glucose (38) or nitrogen (94) limitation. Finally, T7 phage infection of E. coli results in in RelA-dependent (p)ppGpp synthesis (95, 96). Taken together, these examples demonstrate (p)ppGpp synthesis during conditions without explicit amino acid starvation. The role of RelA in all of these examples suggests that changes in amino acid charging are coupled to these cellular transitions, but the molecular mechanisms underlying this coupling are not well understood.

Finally, although the present discussion is limited to RelA-dependent (p)ppGpp synthesis, how does RelA activation relate to the activity of other (p)ppGpp synthases, including SpoT and RelIV (97), in Gram-negative bacteria or nonribosomal synthases (Sas) in Gram-positive bacteria? For example, the RelA product ppGpp is an allosteric activator of the Bacillus subtilis synthase SasB (98), an effect that is important for protein synthesis attenuation in stationary phase (83). Investigation of these and related questions will likely provide new insights into the regulatory network underlying RelA function and further reinforce our understanding of its central role in the bacterial response to nutrient limitation.
